# Sucrose Sensitivity of Honey Bees Is Differently Affected by Dietary Protein and a Neonicotinoid Pesticide

**DOI:** 10.1371/journal.pone.0156584

**Published:** 2016-06-07

**Authors:** Fabien J. Démares, Kendall L. Crous, Christian W. W. Pirk, Susan W. Nicolson, Hannelie Human

**Affiliations:** Social Research Insect Group, Department of Zoology and Entomology, University of Pretoria, Private Bag X20, Hatfield, 0028, Pretoria, South Africa; University of Cologne, GERMANY

## Abstract

Over a decade, declines in honey bee colonies have raised worldwide concerns. Several potentially contributing factors have been investigated, *e*.*g*. parasites, diseases, and pesticides. Neonicotinoid pesticides have received much attention due to their intensive use in crop protection, and their adverse effects on many levels of honey bee physiology led the European Union to ban these compounds. Due to their neuronal target, a receptor expressed throughout the insect nervous system, studies have focused mainly on neuroscience and behaviour. Through the Geometric Framework of nutrition, we investigated effects of the neonicotinoid thiamethoxam on survival, food consumption and sucrose sensitivity of honey bees (*Apis mellifera*). Thiamethoxam did not affect protein and carbohydrate intake, but decreased responses to high concentrations of sucrose. Interestingly, when bees ate fixed unbalanced diets, dietary protein facilitated better sucrose detection. Both thiamethoxam and dietary protein influenced survival. These findings suggest that, in the presence of a pesticide and unbalanced food, honey bee health may be severely challenged. Consequences for foraging efficiency and colony activity, cornerstones of honey bee health, are also discussed.

## Introduction

Bees are essential pollinators, and recently a lot of attention has been given to pollinators, both wild and managed populations. For over a decade, managed colonies of honey bees (*Apis mellifera*) all over the world have been diminished by several factors, *e*.*g*. parasites, diseases, and pesticides [1–4]. All of these are potent threats to honey bee health, either alone or in combination.

Among the main threats, the neonicotinoid pesticides have been the subject of thorough investigations. These eventually led the European Union to promote a renewable three-year ban [5]; this time period was allocated for the manufacturers to give evidence of the harmless effects of their compounds for pollinators. In contrast, after more than 20 years of research, many studies have already reported adverse effects on bees. From colony to individual levels, neonicotinoids at sublethal dosage alter a broad spectrum of behaviours: foraging activity [6]; homing flight and orientation [7]; and learning and memory [8]. At cellular and molecular levels, neonicotinoids target and activate the nicotinic acetylcholine receptor (nAChR) [9]. This receptor is widely distributed in insect nervous systems [10–12], leading many reports to focus primarily on neuroscience and behaviour [8,12,13], but also on other physiological processes, such as sucrose sensitivity [14,15] or midgut toxicity [16].

Recently, effects of pesticides on nutrition have been reported [17]: while honey bees fed with high protein diet resisted the effect of dietary nicotine, the interaction between combined stressors–dietary nicotine, low protein diet and low temperature–impaired survival. Consumption of sugar solutions spiked with neonicotinoids decreased overall food consumption by honey bees but paradoxically, they were attracted to these pesticides [18]. Concomitantly, the same study showed that bees cannot taste neonicotinoids [18]. Using the local honey bee subspecies, *Apis mellifera scutellata*, we previously reported effects of dietary nicotine on honey bees of different life stages. Whereas it did not affect larval survival [19], dietary nicotine differentially affected adult workers, whether by itself or in combination with different stressors such as temperature [17], colony strength [20], diet restriction and immune challenge [21].

Nutrition is the cornerstone to regulating physiological processes, leading to good health [22]. The Geometric Framework of Nutrition [23] explains how any animal, given a combination of unbalanced diets, should regulate its intake to reach an optimum. Through this method, it has become possible to model and test relationships between nutrient intake and different parameters, such as survival, reproductive status [24], collective migration [25] and collective decision-making [26]. Mapping nutrient consumption, classically the ratio of proteins to carbohydrates (P:C), with such parameters allows us to define nutritional landscapes or nutrient space [27,28]. These provide interesting visualisations of different processes within the scope of nutrition, and a strong link between model and experiment [29,30]. Alternatively, it is a good approach for assessing the influence of different stressors, such as pesticides, on nutrition.

In this study, using the Geometric Framework of nutrition approach, we tested how the neonicotinoid thiamethoxam (THX) alters the nutrient intake of newly emerged caged honey bees. First, we tested a range of sublethal dietary doses of THX, as defined in previous studies [16,31,32] (see also Methods section): we used 0.25% LD_50_ (low dose, LOW), 2.5% LD_50_ (medium dose, MED) and 25% LD_50_ (high dose, HIGH) in an experiment where bees were given the choice between two diets differing in P:C ratio. Diets without dietary pesticide were also tested as control treatment (CTRL). This allowed for controlling the pesticide effect alone. In a second experiment, using the same THX doses as previously, bees were restricted to one fixed unbalanced diet (no-choice experiment). We tested four different P:C ratio diets ranging from pure sucrose diet (P:C ratio 0:1) to increasing amounts of protein (P:C ratios 1:100, 1:30 and 1:3). This allowed us to test the effect of the pesticide and dietary protein on different parameters; nutrient intake and survival were monitored for 14 days. Finally, in these two sets of experiments (choice and no-choice), we looked at the effect of THX chronic exposure, as well as dietary protein, on sucrose sensitivity, using the proboscis extension response (PER) assay.

Since honey bees under the influence of neonicotinoid have lower sucrose sensitivity [14] and eat less food [18], we predicted that THX would alter carbohydrate intake and thus decrease survival. Also, since dietary nicotine improves survival when bees are fed with a high protein diet [17], we predicted that the combination of THX dose and dietary protein would affect survival positively, underlying an increase in food intake. For sucrose sensitivity, this is the first time it has been tested twice over 14 days on caged honey bees. Also, the coupled effect of dietary THX and protein has never been investigated before. In addition, because honey bee physiology and behaviour changes over 14 days, as bees transition from nurses to foragers, we predicted that sucrose sensitivity would vary with age and would be affected by dietary THX. Finally, as we work with *Apis mellifera scutellata*, a subspecies relying on a genetically diverse population [33], we expected honey bees to show better resistance to THX than other subspecies.

## Methods

### Animal samplings

Newly-emerged adult honey bees (*Apis mellifera scutellata*) were collected from frames maintained in an incubator at 35°C. Frames were selected from five different colonies housed at the University of Pretoria apiary. The experiments ran from October 2014 to June 2015. After emergence, honey bees were placed in standard hoarding cages (110×85×70mm), with polycarbonate walls, moveable slides, and a wire mesh base for ventilation [34]. Each cage had a plastic sliding frame in front with three small windows allowing the insertion of 15-ml plastic tubes, containing either food or water provided *ad libitum*. Each cage was also provided with a small hanging piece of comb inside for the bees to cluster on. For both choice and no-choice experiments, cages of 120 newly emerged (four mixed colonies) honey bees were established in triplicate, for each diet and each dose of pesticide. In total, 72 cages were used for the overall experiment. Cages were kept in an incubator at 35°C/50% relative humidity, for 15 days.

### Diet and Pesticide Preparation

Honey bees were given diets consisting of different proportions of protein and carbohydrate (P and C) in the form of casein and sucrose, respectively. Agar-based diets were prepared as previously described [17], and blocks of food weighed before use. For each triplicate, cages were provided with either two feeding tubes containing 1:30 and 1:3 P:C ratio diets (Choice experiment), or only one feeding tube containing 0:1 (pure sucrose), 1:100, 1:30 or 1:3 P:C ratio diet (No-choice experiment). For each of these diets, the sucrose concentration corresponds to 66.6%, 65.3%, 63.8% and 50% by mass, respectively [17]. In both experiments, all diets were supplemented with a dose of the neonicotinoid pesticide thiamethoxam (THX). In order to use ecologically relevant doses, LOW, MEDIUM and HIGH doses of THX were chosen, corresponding to 1 ppb, 10 ppb and 100 ppb respectively. These doses are well below the LD_50_ = 4–5 ng/bee [16,31,32], which is equivalent to LC_50_ = 400–500 ppb when average food consumption of 10 mg/bee/day is taken into account. In several studies, THX concentrations have been reported as 0.1–2.9 ppb in the field [35,36], 0.5–11 ppb in nectar [37–39], and 1–95.2 ppb in pollen [35, 37–39]. In 2012, Dively & Kamel reported THX levels of 140–175 ppb in leaves and flowers [38]. Our tested doses are in line with these observations. According to standard practice, acetone was used as an organic solvent for THX; the proportion of acetone in every diet, including CTRL, was lower than 0.05% [8,14].

### Survival and Diet Consumption

These parameters were recorded on a daily basis. Survival of honey bees was monitored; dead bees were counted and removed from cages. At the same time, water and food tubes were renewed. Food left uneaten was frozen at -20°C until the end of the experiment. At the end of the experiment, all tubes containing uneaten food underwent a prolonged drying step at 45°C, lasting 1~2 months. Daily food consumption was calculated as previously described [17] by adding extra empty cages, with water and pre-weighed food tubes, for calibration purposes. The different wet mass blocks of food underwent the same steps of freezing, drying and reweighing, resulting in dry mass blocks. The regression equation determined from the calibration food blocks allowed conversion of initial wet mass into the initial dry mass of each tube used for bee feeding. Subsequently, consumption was determined by subtracting the remaining dry mass of uneaten food from the original dry mass.

### Sucrose Responsiveness of Individual Honey Bees

Honey bees were tested for their responses to sucrose on day 7 and day 14 of both experiments. Sub-samples of 10 honey bees were removed from each cage and kept on ice for 5 min in order to immobilise them. They were then placed in individual Plexiglas holders, fixed with a drop of wax-colophony mixture [40]. Once awake, the honey bee was given 3.0 μl of 50% sucrose solution and placed at 35°C in an incubator to recover from cold anaesthesia. An hour later, honey bees were tested for their water response: for those responding, we allowed them to drink until water satiation was reached. After another hour, honey bees were quickly tested again for their water response, before being tested with increasing sucrose concentrations (0.03%, 0.1%, 0.3%, 1%, 3%, 10% and 30% sucrose). Each concentration was presented to the honey bee with a 1-ml syringe, by touching the antennae. For each concentration, the proboscis extension reflex (PER) of individual honey bees was recorded. Presentations of increasing sucrose concentrations were spaced at intervals of 3 min minimum. Ten minutes after the last sucrose concentration, honey bees were fed with a 50% sucrose solution and simultaneously tested for PER. This step allowed us to check for any habituation process, where the PER is attenuated because of stimuli repetition. Honey bees not responding to any test concentration and feeding solution were discarded from the analysis; in contrast, honey bees responding to all test concentrations and water just before the test were also discarded from analysis. This represents a very small number of bees and was done in order to avoid including bees reacting to stimuli completely unrelated to the sucrose test [8,14].

### Statistics

Survival was analysed using Kaplan-Meier tests. Consumption data were analysed using either One-way ANOVA or Kruskal-Wallis tests, depending on variance homogeneity. Pairwise *post hoc* comparisons were made using Mann-Whitney tests and *p*-values were Bonferroni-corrected accordingly. The sucrose response was also analysed through Kruskal-Wallis and Mann-Whitney tests. Correlations were examined by using the Spearman Correlation test. Statistical *p*-value is considered significant under 0.05. All the statistical tests were done using SPSS 23. Figures and tables were prepared using R 3.2.2, MS Excel 2010 and Photoshop CS2.

## Results

### Survival is influenced by the combination of pesticide and dietary protein

**Fig 1:**
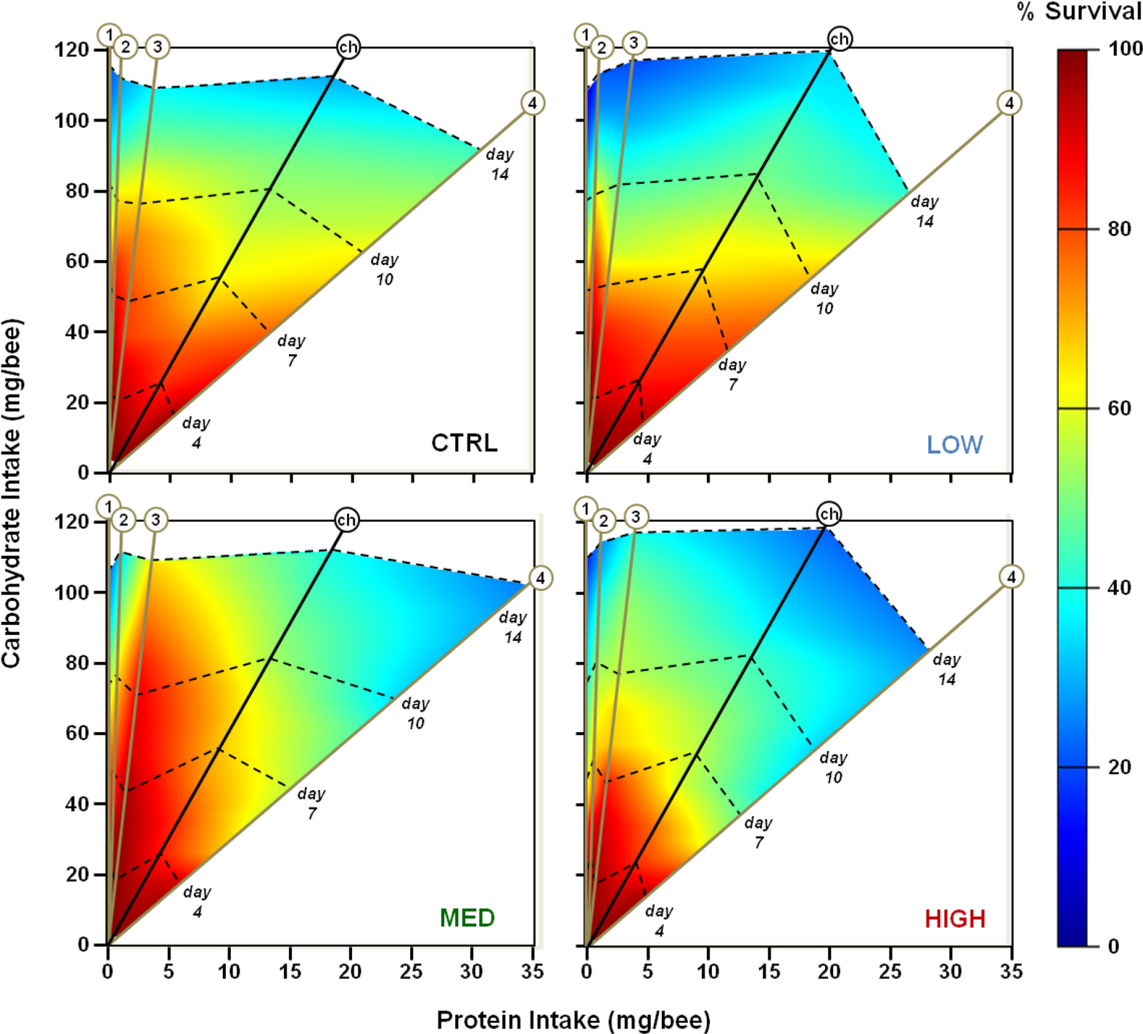
Nutrient intakes and survival of honey bees under the influence of THX. Each panel represents a different THX dose, noted on the bottom-right corner. On each panel, the different rails are the different diets signified by numbers or letters: (1) P:C ratio [0:1]; (2) [1:100]; (3) [1:30]; (4) [1:3]; and (ch), for choice experiment, representing the regulated intake close to the P:C ratio 1:6.5 (see S3 Table). Dotted lines refer to different days, also showing the different intakes and survival rates between diets. For the sake of clarity, we chose to represent only four days (4, 7, 10 and 14). Survival rate is colour-scaled. For values and statistical data, refer to Table 1 and S1 Table.

During the choice experiment, the pesticide significantly affected survival throughout the 14 days of the experiment (LogRank, χ^2^ = 94.435, df = 3, *P*<0.001). At both days 7 and 14, honey bees fed with MED dose had significantly higher survival rates, whereas honey bees fed with HIGH dose had significantly lower survival rates, compared to CTRL and LOW (Table 1 and Fig 1; see also Mann-Whitney tests in S1A Table).

**Table 1:** Survival Rates and Nutrient Consumption at days 7 and 14.

			Survival (%)		Consumption (mg/bee)	
		N	Day 7	Day 14	Day 7	Day 14
**Choice**	**TOTAL**	2880	62.4% ±2.6	31.4% ±3.6	65.3 ±0.8	134.2 ±2.1
	**CTRL**	720	61.7% ±4.4	29.7% ±5.1	64.8 ±1.8	130.3 ±2.6
	**LOW**	720	63.6% ±3.1	33.8% ±8.6	67 ±1.4	138.9 ±4.1
	**MED**	720	*68*.*0% ±6*.*8*	*39*.*7% ±8*.*3*	65 ±1.4	130.1 ±3.9
	**HIGH**	720	56.4% ±5.8	*22*.*4% ±5*.*9*	64.2 ±1.9	137.5 ±5.7
**No-Choice**	**TOTAL**	5781	75.5% ±2.2	29.8% ±2.5	51.5 ±0.8	115.3 ±1.5
	**CTRL**	1442	81.1% ±2.6	32.7% ±4.7	52.3 ±1.1	116.2 ±2.4
	**LOW**	1456	77.9% ±2.5	*22*.*9% ±4*.*6*	52.1 ±1.5	112.7 ±2.7
	**MED**	1447	78.3% ±4.8	36.9% ±5.7	51.2 ±1.9	117.4 ±3.9
	**HIGH**	1436	*64*.*8% ±5*.*8*	*26*.*8% ±4*.*2*	50.4 ±1.7	115 ±3.3
	**[0:1]**	1443	72.5% ±3.6	*16*.*8% ±3*.*3*	50.5 ±1.1	*110*.*3 ±1*.*6*
	**[1:100]**	1448	81.1% ±2.7	*27*.*6% ±2*.*1*	52.2 ±0.9	114 ±1.4
	**[1:30]**	1445	83.1% ±3.3	*41*.*1% ±6*.*1*	50.5 ±1.9	117.2 ±3.7
	**[1:3]**	1445	65.5% ±5.9	*33*.*8% ±4*.*8*	52.8 ±1.9	119.9 ±4.2

Survival rates and nutrient consumption per bee (±s.e.m) after 7 days and 14 days, divided between Choice and No-Choice experiments and subdivided according to THX dose (all diets pooled) or diet (all THX doses pooled). N indicates the sample size of each group at the start of the experiment. Values in italics represent group(s) significantly different from the others. Underlined values show a significant correlation among the different groups (see S1 and S2 Tables for all statistical data). For more details on Choice Experiment protein and carbohydrate intakes, please refer to S3 Table.

Under fixed diets of the no-choice experiment, again the pesticide significantly influenced survival throughout the experiment (LogRank, χ^2^ = 67.696, df = 3, *P*<0.001). When comparing THX doses at day 7, there was a significant correlation between survival and dose: Survival rates decreased as the dietary pesticide dose increased (S2 Table; Spearman coefficient: -0.290, *P* = 0.046). Honey bees fed on HIGH dose had lower survival at day 7 compared to other groups (Table 1; Mann-Whitney test, Z_(HIGH vs others)_ < -7.914, *P*<0.001; see also S1B Table); this is noticeable in Fig 1 by the smaller red area on the HIGH graph, compared to the others. At day 14, MED dose fed honey bees had a significantly higher survival rate compared to other groups (Table 1; Mann-Whitney test, Z_(MED vs others)_ < -2.086, *P*<0.037 maximum; see also S1B Table).

Diet also significantly influenced survival (LogRank, χ^2^ = 166.477, df = 3, *P*<0.001). On day 7, honey bees fed with pure-sucrose diet (P:C 0:1) and high-protein diet (P:C 1:3) had significantly lower survival rates compared to intermediate protein diets (P:C 1:100 and 1:30, respectively) (Table 1; see also S1C Table). On day 14, honey bees fed with pure-sucrose diet had the significantly lowest survival rate, compared to those fed on the protein diets (Table 1; Mann-Whitney test, Z_(0:1 vs others)_ < -5.860, *P*<0.001; see also S1C Table). Also, there was a significant correlation between survival and diet: survival increased with increasing protein concentration (S2 Table; Spearman coefficient: 0.449, *P* = 0.001).

### Cumulative consumption differs between diets, but is not influenced by pesticide

When given the choice between diets, honey bees consumed similar amounts of food, independent of THX doses (Table 1); the cumulative intake was not different between groups at day 7 or day 14 (S1A Table; One-way ANOVA; Day 7: F_(3, 20)_ = 0.546, *P* = 0.657; Day 14: F_(3, 20)_ = 1.203, *P* = 0.334). Moreover, all honey bees settled at a preferred regulated intake target close to P:C ratio 1:6.5, again independent of dietary THX (S3 Table).

During the no-choice experiment, as above, the comparison of THX doses revealed no difference between groups on days 7 and 14, when it came to cumulative food intake (S1B Table; One-way ANOVA; Day 7: F_(3, 44)_ = 0.315, *P* = 0.815; Day 14: F_(3, 44)_ = 0.512, *P* = 0.676).

When focusing on fixed diets, honey bees consumed similar amounts of food on day 7 (Table 1; One-way ANOVA; Day 7: F_(3, 44)_ = 0.659, *P* = 0.582). However, on day 14, there was an influence of the diets on overall consumption (Kruskal-Wallis, χ^2^ = 8.445, df = 3, *P* = 0.037). The honey bees ate significantly less of the pure sucrose diets than the other diets (Table 1; Mann-Whitney test, Z_(0:1 vs others)_ < -2.136, *P*<0.04 maximum; see also S1B Table) and there was a significant positive correlation between cumulative food intake and dietary protein concentration (S2 Table; Spearman coefficient: 0.390, *P* = 0.006). While not significantly different, daily nutrient intake also reflected this trend with a distinct consumption pattern differing between low protein diets and higher protein diets, the latter showing a pattern close to what happened in the choice experiment (S1 Fig).

### Both pesticide and dietary protein alter sucrose response, but differently

**Fig 2:**
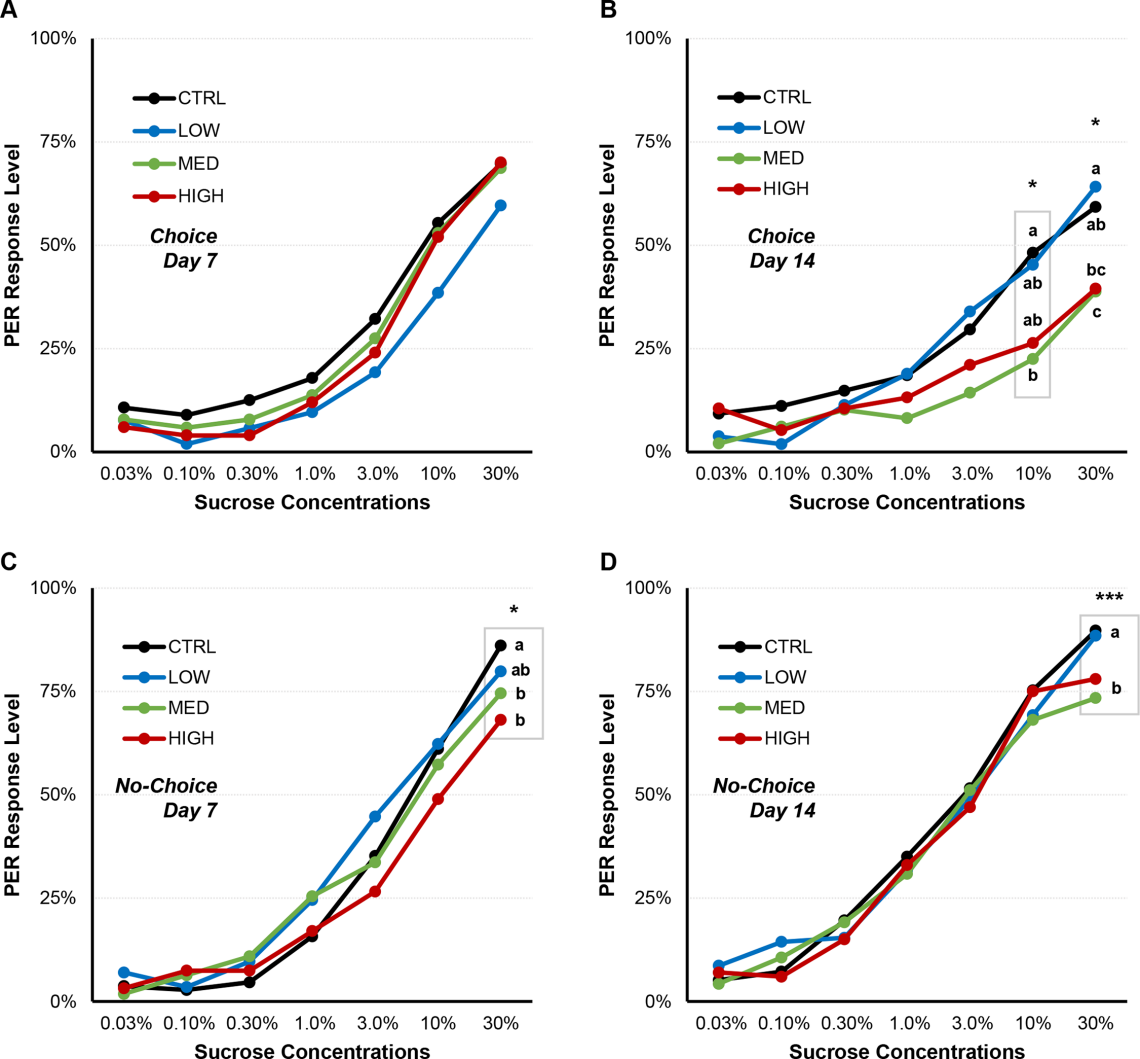
PER response levels to different sucrose concentrations and influence of THX. During choice experiment, THX had no effect at day 7 (**A**) and influenced PER response to high sucrose concentrations at day 14 (**B**), with lower levels for medium and high doses of THX. During no-choice experiment, THX influenced PER response to high sucrose concentrations at both day 7 (**C**) and day 14 (**D**), again with lower levels for medium and high doses of THX. Kruskal-Wallis significance (* p<0.05; ** p<0.01; *** p<0.005) indicates where PER response are influenced by THX. Letters represent post hoc Mann-Whitney pairwise comparisons, and different letters mean significant differences between THX groups. Grey boxes represent a significant correlation between groups. Refer to Table 2 for related PER values. For all statistical data, refer to S1 Table.

During the choice experiment, on day 7, THX did not significantly affect sucrose response levels, compared to the control (Fig 2A; see also Kruskal-Wallis analyses in S1A Table). However on day 14, the response to sucrose was altered by the pesticide, especially responses to 10% and 30% sucrose (Fig 2B; 10% sucrose: Kruskal-Wallis, χ^2^ = 10.371, df = 3, *P* = 0.013; 30% sucrose: Kruskal-Wallis, χ^2^ = 10.023, df = 3, *P* = 0.018). Honey bees fed with MED and HIGH doses had significantly lower PER levels compared to CTRL and LOW for both sucrose concentrations (Table 2 & Fig 2; see also Mann-Whitney analyses in S1A Table).

**Table 2:** Response Levels to 10% and 30% Sucrose Concentrations.

PER		Day 7			Day 14		
		N	10%	30%	N	10%	30%
**Choice**	**CTRL**	56	55.4%	69.6%	54	*48*.*1%*	59.3%
	**LOW**	52	38.5%	59.6%	53	*45*.*3%*	64.2%
	**MED**	51	52.9%	68.6%	49	*22*.*4%*	*38*.*8%*
	**HIGH**	50	52.0%	70.0%	38	*26*.*3%*	*39*.*5%*
**No-Choice**	**CTRL**	108	61.1%	86.1%	97	75.3%	89.7%
	**LOW**	114	62.3%	79.8%	104	69.2%	88.5%
	**MED**	110	57.3%	74.5%	94	68.1%	*73*.*4%*
	**HIGH**	94	48.9%	*68*.*1%*	100	75.0%	*78*.*0%*

Sucrose response levels at days 7 and 14, divided between Choice and No-Choice experiments and subdivided by doses (all diets pooled). N indicates the sample size of each group at the start of the experiment. Values in italics represent group(s) significantly different from the others. Underlined values show a significant correlation among the different groups: the higher the THX dose, the lower the response levels (see S1 and S2 Tables for all statistical data).

When honey bees were fed fixed diets, on days 7 and 14 (Fig 2C & 2D), THX had a significant effect only on the 30% sucrose response (All diets combined—Kruskal-Wallis; Day 7: χ^2^ = 10.239, df = 3, *P* = 0.017; Day 14: χ^2^ = 12.809, df = 3, *P* = 0.005). The honey bees fed with MED and HIGH diets on day 7 had significantly lower PER levels compared to CTRL (Table 2; see also Mann-Whitney analyses in S1B Table). On day 14, honey bees fed with MED and HIGH diets showed significantly lower PER levels than CTRL and LOW (Table 2; see also Mann-Whitney analyses in S1B Table). The pesticide dose was negatively correlated to sucrose response both at days 7 and 14: the higher the dose, the lower the response to high sucrose concentration (S2 Table; Day 7: Spearman coefficient: -0.351, *P* = 0.016; Day 14: Spearman coefficient: -0.439, *P* = 0.002).

**Fig 3:**
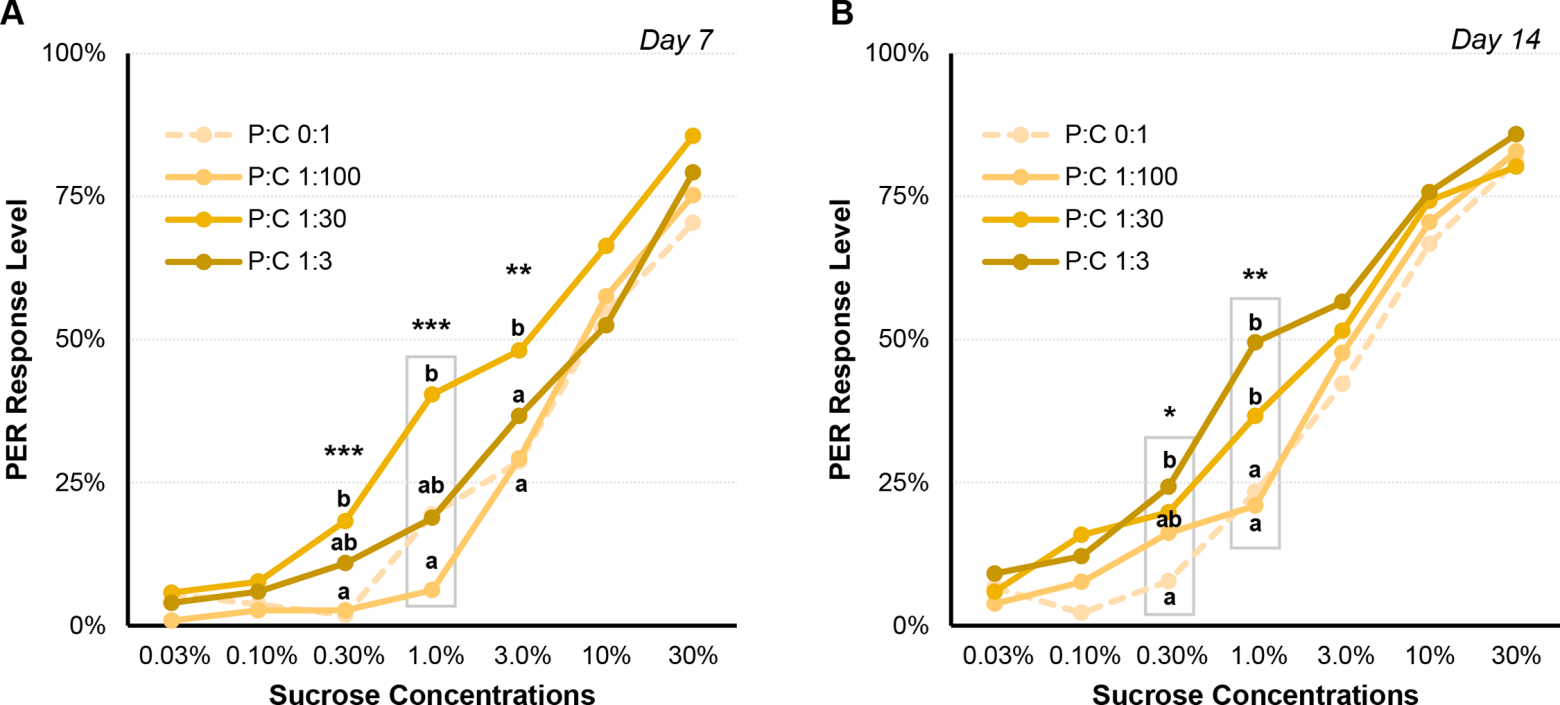
PER response levels to different sucrose concentrations and influence of dietary protein. During the no-choice experiment, the dietary protein from the different P:C ratio diets affects PER response to lower sucrose concentrations at both day 7 (**A**) and day 14 (**B**): the higher the dietary protein concentration in diet, the higher the PER response rate. Kruskal-Wallis significance (* p<0.05; ** p<0.01; *** p<0.005) indicates where PER response are influenced by dietary protein. Letters represent post hoc Mann-Whitney pairwise comparisons, and different letters mean significant differences between dietary protein groups. Grey boxes represent a significant correlation between groups. Refer to Table 3 for related PER values. For all statistical data, refer to S1 Table.

When fed on fixed diets, on day 7, honey bees showed altered responses to intermediate sucrose concentrations: 0.3%, 1% and 3% sucrose (All pesticide doses combined—Fig 3A; Kruskal-Wallis; 0.3%: χ^2^ = 25.273, df = 3, *P*<0.001; 1.0%: χ^2^ = 38.988, df = 3, *P*<0.001; 3.0%: χ^2^ = 11.357, df = 3, *P* = 0.010). Honey bees fed with P:C 1:30 diets showed significantly higher response levels to compared to other groups (Table 3, Fig 3A; see also Mann-Whitney analyses in S1C Table). On day 14, response levels were significantly different only for 0.3% and 1% sucrose (Kruskal-Wallis; 0.3%: χ^2^ = 9.582, df = 3, *P* = 0.022; 1.0%: χ^2^ = 23.527, df = 3, *P*<0.001). In these cases, honey bees fed with either P:C 1:30 or 1:3 diets responded significantly more compared to the two other diets (Table 3, Fig 3B; see also Mann-Whitney analyses in S1B Table). On day 14 as well, there was a significant positive correlation between dietary protein and response to intermediate sucrose concentration: the higher the protein, the higher the response rate (Table 3; S2 Table; 0.30%: Spearman coefficient: 0.378, *P* = 0.008; 1.0%: Spearman coefficient: 0.442, *P* = 0.002).

**Table 3:** Response Levels to Low Sucrose Concentrations.

PER	Day 7				Day 14			
	N	0.30%	1.0%	3.0%	N	0.30%	1.0%	3.0%
**Choice**	209	7.7%	13.4%	25.8%	194	11.9%	14.9%	25.3%
**No-Choice**	426	8.2%	20.9%	35.4%	395	17.2%	32.7%	49.6%
**[0:1]**	108	1.9%	19.4%	28.7%	90	7.8%	23.3%	42.2%
**[1:100]**	113	2.7%	*6*.*2%*	29.2%	105	16.2%	21.0%	47.6%
**[1:30]**	104	*18*.*3%*	*40*.*4%*	*48*.*1%*	101	19.8%	36.6%	51.5%
**[1:3]**	101	*10*.*9%*	18.8%	36.6%	99	*24*.*2%*	*49*.*5%*	56.6%

Sucrose response levels at days 7 and 14, divided between Choice and No-Choice experiments and subdivided by diet (all THX doses pooled). N indicates the sample size of each group at the start of the experiment. Values in italics represent group(s) significantly different from the others. Underlined values show a significant correlation among the different groups: the higher the dietary protein concentration, the higher the response level (see S1 and S2 Tables for all statistical data).

## Discussion

The aim of this study was to observe the influence of thiamethoxam upon nutrition in honey bees and how it affects survival, food consumption and sucrose sensitivity. We found that, independent of the THX dose, survival is higher on balanced diets, *i*.*e*. when honey bees have a choice and are able to regulate their optimum nutrient intake. With fixed unbalanced diets as in the no-choice experiment, honey bees require protein to survive better [22], but too much protein is detrimental to survival [17,41]. In our study, THX also influenced survival, negatively at the high dose, and positively at the medium one. However, this THX effect was only seen with balanced diets and with the fixed P:C 1:30 diet. When restricted to the P:C 1:3 diet with higher protein, honey bees with medium and high dietary THX doses showed reduced survival. Similar results have been observed, depending on the colony strength [20], with dietary nicotine: when honey bees came from a strong colony, dietary nicotine decreased survival, whereas bees from a weak colony survived better when given the same dose of dietary nicotine. This might underline a potent interaction between protein and THX, making honey bees not able to cope with this interactive effect.

On the other hand, inclusion of THX in the diet did not affect nutrient consumption in any way. Honey bees were able to regulate their food intake correctly even under pesticide stress. When given fixed unbalanced diets, the amount of food consumed was also no different between control and THX groups (Table 1). Whatever process is used by the honey bees in order to regulate their P:C intake, THX does not affect it. In contrast, dietary proteins influenced consumption in a positive direction: the higher the protein concentration, the more diet was eaten (S1 Fig). This reflects a compensation phenomenon, with bees trying to reach the optimum carbohydrate intake. Noticeably, this shift towards carbohydrates has been a common feature in previous studies, with *A*. *m*. *scutellata* honey bees fed on solid diets with casein or other protein sources [17,42], and with *A*.*mellifera* Buckfast hybrid honey bees and *Bombus terrestris* bumblebees fed on liquid diets containing essential amino acids [43,44]. These reports together with the present study show that bees choose to prioritise carbohydrate intake over protein/amino acid intake.

More interesting, thanks to chemical similarities between nicotine and neonicotinoids, we can compare our results with the effect of dietary nicotine [17]. Nicotine and THX do not change nutrient regulation, regardless of the dietary dose: when given the choice, honey bees regulate their consumption toward a similar optimal P:C ratio close to 1:6.5 (see S3 Table). Also, both nicotine and THX lead to an increase in survival with specific combinations of pesticide doses and dietary protein. This can be referred to as ‘stress response hormesis’ [17], where a mild stress triggers cascade reactions leading to increased energetic investment in somatic maintenance, eventually improving life span [45,46].

**Fig 4:**
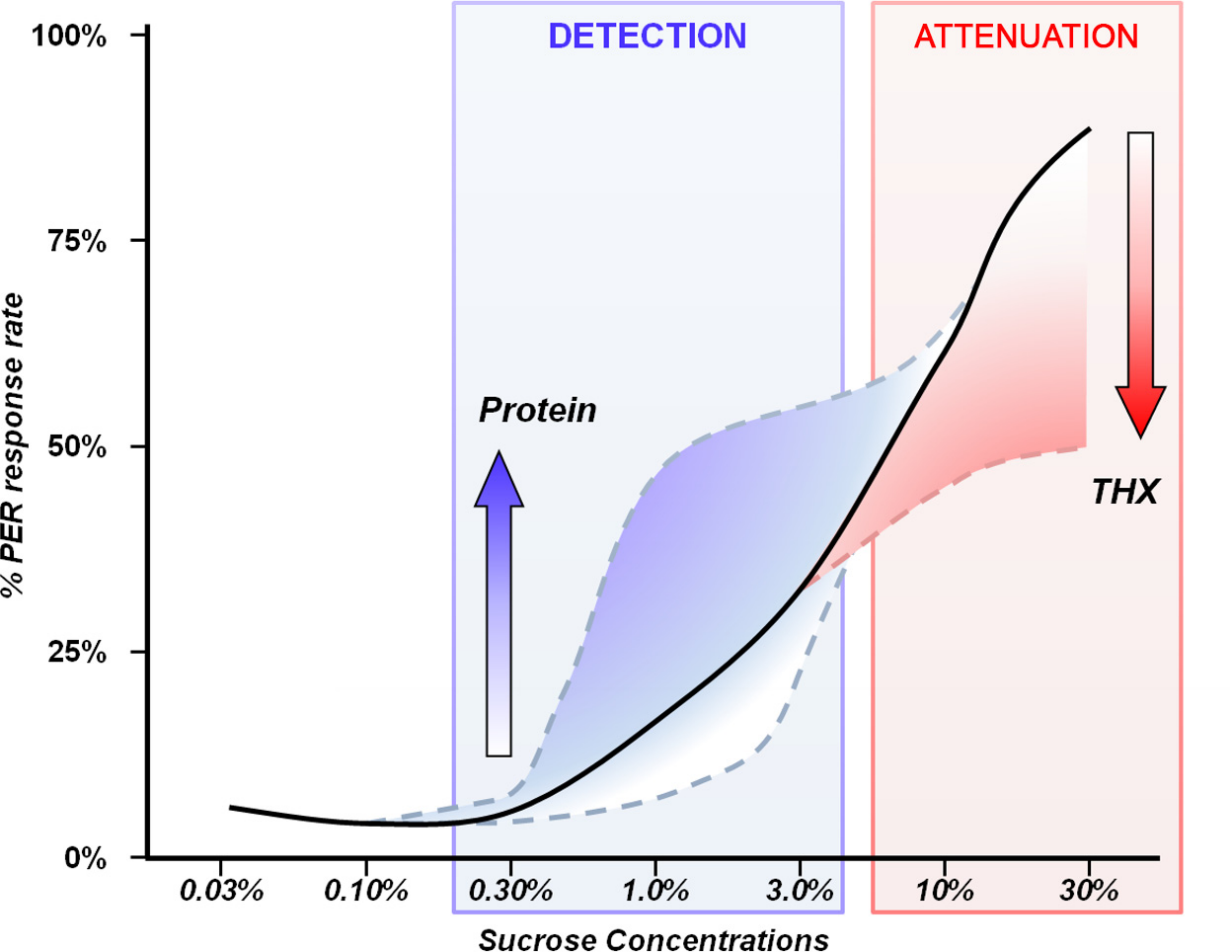
Schematic diagram of the effects of dietary protein and THX on sucrose sensitivity. Both protein and pesticide affect sucrose sensitivity, but not at the same levels, and in opposite directions. Consider the plain line as the regular expected response to sucrose from control bees. When dietary protein concentration increases, the sucrose sensitivity increases too. Whereas the first detectable sucrose concentrations (0.30%–1%) elicit a very low response rate in control bees, dietary protein increases their response rate by improving detection of these low sucrose concentrations. In contrast, when dietary THX dose increases, the sucrose sensitivity decreases. Acting on higher sucrose concentrations (10%–30%), dietary THX impedes the normal rate of response compared to control bees, leading to taste attenuation.

Other significant results came from the PER sucrose sensitivity tests, where both dietary protein and THX altered sucrose response (Fig 4). Few studies have looked at the effect of age and genotype on sucrose threshold of bees directly collected from the hive [47–50]. We monitored sucrose sensitivity twice over the course of two weeks on caged honey bees, and were thus able to assess variation of sucrose response with worker bee age in a controlled environment. Moreover, even though it is known that pollen foragers have a higher sucrose sensitivity compared to nectar foragers [48], the effect of dietary protein on sucrose sensitivity in caged honey bees has never been reported until now. We observed higher response levels to intermediate concentrations of sucrose (0.30% to 3%) correlated with higher dietary protein concentrations (Fig 3). It seems that dietary protein may facilitate better sucrose detection. One explanation could be that liquid sugar solutions given during the PER tests taste different from the solid diets with proteins that the bees have been consuming; the more protein in the diet, the more different the taste. Honey bees of different patrilines [49] are destined to become either pollen foragers, with lower sucrose thresholds, or nectar foragers [50]. Here, independent of genotype, we hypothesize that honey bee pollen foraging ontogeny may also be influenced by dietary protein.

In contrast with the dietary protein effect on PER, medium and high doses of THX led to decreased response levels to high concentrations of sucrose over time (10% and 30% sucrose, Fig 2A and 2B). The pesticide attenuates the response to high concentrations of sucrose, and bees treated with high doses of pesticides will require higher concentrations of sucrose to be stimulated. Although a partial decrease in sucrose sensitivity caused by THX has been previously reported [14] the sucrose response was monitored only once after 11 days. Here, we report a progressive effect of THX altering sucrose sensitivity, under chronic exposure for 14 days; this may indicate a progressive attenuation of taste. Recently it was reported that neonicotinoids do not elicit activation of gustatory receptors on basiconic sensillae [18], implying that bees cannot taste neonicotinoids; but nAChRs are present in the sub-oesophageal ganglion, a brain region receiving sensory neurons mediating taste stimuli [51]. For instance, the neuron VUMmx1 is known to mediate the unconditioned stimulus information [52], *i*.*e*. the sucrose response used in the PER classical conditioning [53, 54]. This neuron projects toward glomeruli of the antennal lobes and Kenyon cells of the mushroom bodies [55] and releases octopamine on the post-synaptic elements of the projection neurons [53]. These projection neurons and Kenyon cells also express nAChRs [56–58]. Thus, if neonicotinoids saturate or desensitize nAChRs, activity of projection neurons and Kenyon cells will not be efficiently modulated by the VUMmx1 firing, regardless of the strength of sucrose stimulation. In other words, the sucrose signal can be affected downstream of the VUMmx1 neuron. This could be an alternate hypothesis to taste attenuation and/or absence of taste.

Together, these two results–increased detection through dietary protein and decreased sensitivity through the neonicotinoid effect–bring up the question of the influence of THX and other neonicotinoids on the fate of pollen foragers and the consequent effects on brood development inside the colony. Indeed, whereas honey bees with higher sucrose sensitivity become pollen foragers [50], the remaining bees will collect nectar. Under chronic exposure of neonicotinoids, honey bees will experience a decrease in their sucrose sensitivity: we make the assumption that nurses influenced by THX inside the hive may be fated to become nectar foragers (and not efficient nectar foragers, in addition). This could lead to less pollen brought back to the hive together with nectar of less-than-optimal quality. This may indirectly affect brood feeding and eventually brood development. So far, no study has explored the influence of brood pheromone on pesticide-exposed bees to see whether they would be able to forage for pollen.

Alteration of sucrose sensitivity through time may reflect how processing of THX, and pesticides more generally, can vary depending on honey bee age. In our study, we observed that the effect of THX on sucrose sensitivity became more pronounced after 14 days. A possible explanation could be that the energy cost related to pesticide detoxification may be greater during the first week of age, leading to precocious energy depletion and death, whereas honey bees in the second week may be the ones spending sufficient energy to counteract the first-week pesticide effect and survive, only to be affected ultimately through other relevant physiological processes, one being sucrose sensitivity.

Furthermore, understanding the whole repertoire of responses to neonicotinoid exposure or other pesticides should be done with highly diverse populations which are mostly unaffected by artificial selection processes. We used the local honey bee subspecies, *Apis mellifera scutellata*, while most of the studies conducted in the northern hemisphere were done using *A*. *m*. *mellifera* or *A*. *m*. *ligustica*, or a breed of the latter two. Even though they belong to the same species and share the same genome, the *A*. *m*. *scutellata* subspecies differs in its management: the population is mainly wild, compared to northern honey bees which are mainly, if not completely, managed populations [59,60]. The minority of managed *A*. *m*. *scutellata* hives may rely on a vast pool of wild colonies, making it a genetically diverse population [33]; hence it is interesting to consider the effects of neonicotinoids on this subspecies. Being more diverse, less selected for docility and mainly driven by natural selection in a wild environment, we would expect this population to show either resistance or higher sensitivity to these pesticides, compared to northern managed and artificially selected subspecies. Our study tends to indicate the *A*. *m*. *scutellata* subspecies behaves and reacts in the same range as northern managed populations, but more studies are required to settle this argument.

The detoxification process happening in the presence of dietary nicotine was recently described in newly emerged *A*. *m*. *scutellata* [61]: metabolic resistance, actively detoxifying nicotine, leads to an increase in energetic cost, antioxidant production and heat shock stress response. Interestingly, it was suggested that nicotine tolerance may vary depending on age, based on the fact that foragers express less antioxidants and enzymes involved in detoxification [62,63]. In parallel, the neonicotinoid imidacloprid reduces mitochondrial activity of Africanized honey bees, depleting the ATP available [64]. Therefore, one side-effect of nicotine and neonicotinoids is the impact on cellular energy production, in addition to their action on insect nervous systems. In our study, we can make the assumption that the right THX dose might trigger the detoxification process, whereas the right dietary protein concentration may regulate this same process. In the other hand, too much protein and THX would affect the triggering and regulation of the detoxification process and might impair mitochondrial activity; this eventually leads to reduced energy available for the honey bee and ultimately, a decrease in behavioural activities.

The PER sucrose assay is related to nectar foraging behaviour [54,65] and results from this assay can give insights to what may happen when bees visit flowers [50]. In our study, the neonicotinoid impaired responses to high sucrose concentrations (Figs 2 and 4). It means that honey bees chronically exposed to the pesticide would show diminished responses to nectar; it would require higher sugar concentrations in nectar to stimulate the honey bees and less nectar might be brought back into the hive. That may be an additional explanation for the sublethal effect of neonicotinoids on foraging behaviour reported previously [6,7] with longer foraging trips and failure to get back to the hive. Results from our study showed a decrease in sucrose sensitivity correlated to dietary THX dose. Together with possible energy depletion, this could mean that poisoned honey bees spend more time and energy trying to find concentrated nectar (and probably failing to do so). A very recent report points toward that possibility, showing how bumblebees poisoned with THX increased their frequency of visiting apple flowers but without providing better pollination service at an individual level [66].

Foraging for pollen and nectar is crucial for colony activity and pollination [67,68]; slight impairments of nectar foraging may have great consequences on the overall nutrition status of the colony, and consequently on colony health [22]. We reported a positive effect of a medium THX dose, coupled with a balanced diet, on survival. Our low and medium doses correspond to field-realistic concentrations of thiamethoxam reported so far [35–39]. But it is unlikely that bees will often encounter such a benign situation in the environment. Managed populations of honey bees are usually not far from pesticide-treated crops [31,36,69]. Also, monofloral crops are the equivalent of fixed unbalanced diets and might actually act as threat enhancers to honey bee health [4,60]. In order to protect hives, beekeeping practices must be adapted to local conditions and provide different protein sources to colonies when applicable.

## Supporting Information

S1 Fig Daily nutrient intake is influenced by the amount of dietary protein, but not by the THX pesticide dose. The panels show the effect of THX doses among different diets. The top panel is the choice experiment, where bees were able to regulate their nutrient intake. The four panels below the line represent the no-choice experiment with the four different fixed diets (indicated at the top-left corner of each panel). For each panel, days are indicated on the x-axis, from day 1 to day 14, while the four THX doses are represented on the y-axis. Nutrient intake (in mg/bee) is colour-scaled. Maximal intake is achieved during the choice experiment, around day 6, independent of THX dose. The patterns of daily consumption of P:C ratios 1:30 and 1:3 diets are similar to those in the choice experiment, although the maximum intake is around day 7. In contrast, when dietary protein is low or absent, daily consumption is reduced, especially on the second week.
(PDF)

S1 Table Statistical data. (A) Choice experiment. (B) No-Choice experiment–THX Dose effect. (C) No-Choice experiment–Dietary protein effect.
(PDF)

S2 Table Spearman correlations. (PDF)

S3 Table Cumulative consumption and details of the different amount of nutrient eaten during the Choice Experiment. Every values are in mg/bee ±s.e.m., except for the P:C ratio columns. The “Cumulative Consumption” column is the same as the last column in Table 1. Honey bees were offered the choice between two unbalanced diets, differing in their P:C ratios (1:3 and 1:30). First, consumption of each diet is assessed and proportions of protein and carbohydrate are calculated. The total protein consumption is the addition of the protein parts eaten from the 1:3 diet and the 1:30 diet; the same goes for the carbohydrate part. P:C ratios are calculated by dividing the total protein consumption by the total carbohydrate consumption.
(PDF)

## Abbreviations

THX: Thiamethoxam
CTRL: control
MED: medium
nAChR: nicotinic acetylcholine receptor
P:C ratio: protein to carbohydrate ratio
LD_50_: lethal dose 50%
