# Supplementary material for: Sucrose Sensitivity of Honey Bees Is Differently Affected by Dietary Protein and a Neonicotinoid Pesticide
Source: PLoS One. 2016 Jun 7;11(6):e0156584. doi: 10.1371/journal.pone.0156584 (PMC4896446; doi:10.1371/journal.pone.0156584)
Supplement: S1 Fig — The panels show the effect of THX doses among different diets. The top panel is the choice experiment, where bees were able to regulate their nutrient intake. The four panels below the line represent the no-choice experiment with the four different fixed diets (indicated at the top-left corner of each panel). For each panel, days are indicated on the x-axis, from day 1 to day 14, while the four THX doses are represented on the y-axis. Nutrient intake (in mg/bee) is colour-scaled. Maximal intake is achieved during the choice experiment, around day 6, independent of THX dose. The patterns of daily consumption of P:C ratios 1:30 and 1:3 diets are similar to those in the choice experiment, although the maximum intake is around day 7. In contrast, when dietary protein is low or absent, daily consumption is reduced, especially on the second week. (PDF) [file pone.0156584.s001.pdf]

S1 Figure

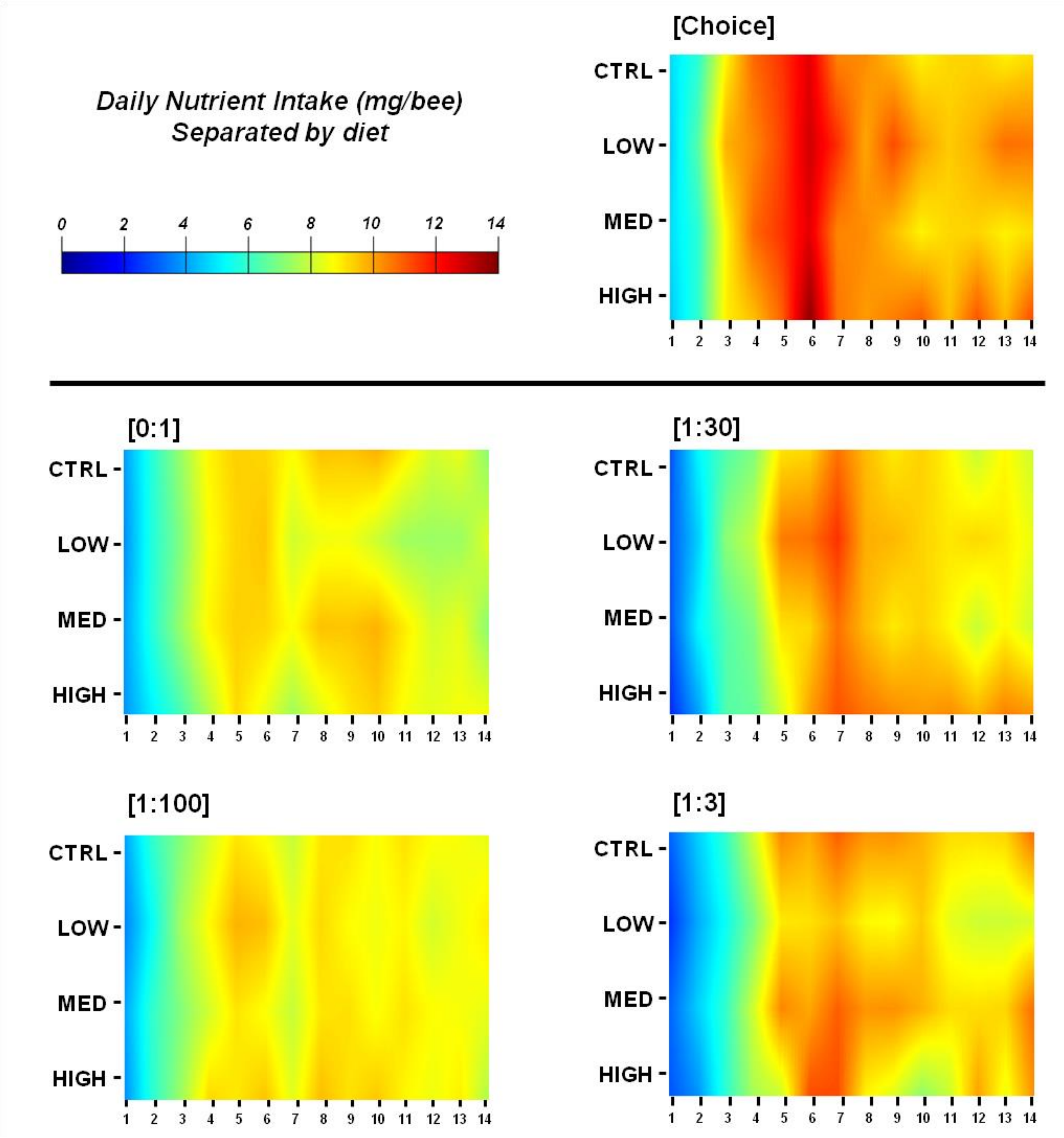

S1 Fig. Daily nutrient intake is influenced by the amount of dietary protein, but not by the THX pesticide dose. The panels show the effect of THX doses among different diets. The top panel is the choice experiment, where bees were able to regulate their nutrient intake. The four panels below the line represent the no-choice experiment with the four different fixed diets (indicated at the top-left corner of each panel). For each panel, days are indicated on the x-axis, from day 1 to day 14, while the four THX doses are represented on the y-axis. Nutrient intake (in mg/bee) is colour-scaled. Maximal intake is achieved during the choice experiment, around day 6, independent of THX dose. The patterns of daily consumption of P:C ratios 1:30 and 1:3 diets are similar to those in the choice experiment, although the maximum intake is around day 7. In contrast, when dietary protein is low or absent, daily consumption is reduced, especially on the second week.
