# Supplementary material for: Sucrose Sensitivity of Honey Bees Is Differently Affected by Dietary Protein and a Neonicotinoid Pesticide
Source: PLoS One. 2016 Jun 7;11(6):e0156584. doi: 10.1371/journal.pone.0156584 (PMC4896446; doi:10.1371/journal.pone.0156584)
Supplement: S1 Table — (A) Choice experiment. (B) No-Choice experiment–THX Dose effect. (C) No-Choice experiment–Dietary protein effect. (PDF) [file pone.0156584.s002.pdf]

S1 Table. (A) Choice Experiment

(1) SURVIVAL

Kaplan-Meier Analyses

Overall Comparisons				
	Chi-Square	df	*P-value*	
Log Rank (Mantel-Cox)	94.435	3	***<0.001***	***

Test of equality of survival distributions for the different levels of Dose.

Pairwise Comparisons					
	Compared Doses		Chi-Square	*P*	
Log Rank (Mantel-Cox)	CTRL	LOW	0.095	0.757	
	CTRL	MED	42.033	***<0.001***	***
	CTRL	HIGH	11.253	***0.001***	***
	LOW	MED	37.929	***<0.001***	***
	LOW	HIGH	12.072	***0.001***	***
	MED	HIGH	94.599	***<0.001***	***

Mann-Whitney Comparisons

Day 7		Mann-Whitney U			
Compared Doses		MW-U	Z	*P-value*	
CTRL	LOW	255240.0	-0.583	0.560	
CTRL	MED	235440.0	-3.566	***<0.001***	***
CTRL	HIGH	246600.0	-1.850	0.064	
LOW	MED	231480.0	-4.147	***<0.001***	***
LOW	HIGH	250560.0	-1.267	0.205	
MED	HIGH	222840.0	-5.403	***<0.001***	***

Day 14		Mann-Whitney U			
Compared Doses		MW-U	Z	*P-value*	
CTRL	LOW	252000.0	-1.166	0.244	
CTRL	MED	232920.0	-4.113	***<0.001***	***
CTRL	HIGH	241920.0	-2.965	***0.003***	***
LOW	MED	240120.0	-2.954	***0.003***	***
LOW	HIGH	234720.0	-4.122	***<0.001***	***
MED	HIGH	215640.0	-7.028	***<0.001***	***

S1 Table. (A) Choice Experiment

(2) NUTRITION

One-Way ANOVA

Test of Homogeneity of Variances				
**Variables**	**Levene Stat**	**df1**	**df2**	***P-value***
Day 7	0.347	3	20	0.792
Day 14	1.472	3	20	0.252

ANOVA						
**Variables**		**Σ Squares**	**df**	**Mean Square**	**F**	***P-value***
Day 7	Between Groups	26.832	3	8.944	0.546	0.657
	Within Groups	327.635	20	16.382		
	Total	354.467	23			
Day 14	Between Groups	388.202	3	129.401	1.203	0.334
	Within Groups	2152.084	20	107.604		
	Total	2540.287	23			

S1 Table. (A) Choice Experiment

(3) SUCROSE RESPONSE

Kruskal-Wallis Analyses

Non-Parametric Overall Comparison

Variables		Chi-Square	df	*P-value*	
Day 7	0.03%	0.819	3	0.845	
	0.10%	2.858	3	0.414	
	0.30%	3.054	3	0.383	
	1.0%	1.682	3	0.641	
	3.0%	2.492	3	0.477	
	10%	3.646	3	0.302	
	30%	1.715	3	0.634	

Variables		Chi-Square	df	*P-value*	
Day 14	0.03%	4.074	3	0.254	
	0.10%	3.981	3	0.264	
	0.30%	0.656	3	0.884	
	1.0%	3.036	3	0.386	
	3.0%	6.123	3	0.106	
	10%	10.731	3	***0.013***	*
	30%	10.023	3	***0.018***	*

Post-hoc Analyses

Non-Parametric Pairwise Comparisons

Day 14 - Sucrose 10%		Mann-Whitney U			
Compared Doses		MW-U	Z	*P-value*	
CTRL	LOW	1390.0	-0.296	0.768	
CTRL	MED	983.0	-2.702	***0.007***	**
CTRL	HIGH	802.0	-2.101	***0.036***	*
LOW	MED	1002.0	-2.415	***0.016***	*
LOW	HIGH	816.0	-1.834	0.067	
MED	HIGH	895.0	-0.416	0.678	

Day 14 - Sucrose 30%		Mann-Whitney U			
Compared Doses		MW-U	Z	*P-value*	
CTRL	LOW	1361.0	-0.518	0.604	
CTRL	MED	1052.0	-2.066	***0.039***	*
CTRL	HIGH	823.0	-1.859	0.063	
LOW	MED	969.0	-2.550	***0.011***	*
LOW	HIGH	758.5	-2.316	***0.021***	*
MED	HIGH	924.5	-0.066	0.948	

S1 Table. (B) No-Choice Experiment – data separated by THX doses (all diets pooled)

(1) SURVIVAL

Kaplan-Meier Analyses

Overall Comparisons				
	Chi-Square	df	*P-value*	
Log Rank (Mantel-Cox)	67.696	3	***<0.001***	***

Test of equality of survival distributions for the different levels of Dose.

Pairwise Comparisons					
	Compared Doses		Chi-Square	*P-value*	
Log Rank (Mantel-Cox)	CTRL	LOW	25.433	***<0.001***	***
	CTRL	MED	2.419	0.120	
	CTRL	HIGH	25.410	***<0.001***	***
	LOW	MED	42.028	***<0.001***	***
	LOW	HIGH	0.222	0.637	
	MED	HIGH	39.156	***<0.001***	***

Mann-Whitney Comparisons

Day 7		Mann-Whitney U			
Compared Doses		MW-U	Z	*P-value*	
CTRL	LOW	1014909.0	-2.213	***0.027***	*
CTRL	MED	1013685.0	-1.896	0.058	
CTRL	HIGH	862942.0	-10.040	***<0.001***	***
LOW	MED	1048317.5	-0.315	0.753	
LOW	HIGH	906042.0	-7.914	***<0.001***	***
MED	HIGH	895413.0	-8.208	***<0.001***	***

Day 14		Mann-Whitney U			
Compared Doses		MW-U	Z	*P-value*	
CTRL	LOW	954338.0	-5.173	***<0.001***	***
CTRL	MED	1003572.5	-2.086	***0.037***	*
CTRL	HIGH	980056.0	-2.990	***0.003***	***
LOW	MED	917547.0	-7.252	***<0.001***	***
LOW	HIGH	1006204.0	-2.178	***0.029***	*
MED	HIGH	943905.0	-5.069	***<0.001***	***

S1 Table. (B) No-Choice Experiment – data separated by THX doses (all diets pooled)

(2) NUTRITION

One-Way ANOVA

Test of Homogeneity of Variances

Variables	Levene Stat	df1	df2	*P-value*
Day 7	0.763	3	44	0.521
Day 14	2.297	3	44	0.091

ANOVA

Variables		Σ Squares	df	Mean Square	F	*P-value*
Day 7	Between Groups	27.544	3	9.181	0.315	0.815
	Within Groups	1284.317	44	29.189		
	Total	1311.861	47			
Day 14	Between Groups	140.985	3	46.995	0.512	0.676
	Within Groups	4041.385	44	91.850		
	Total	4182.371	47			

S1 Table. (B) No-Choice Experiment – data separated by THX doses (all diets pooled)

(3) SUCROSE RESPONSE

Kruskal-Wallis Analyses

Non-Parametric Overall Comparison					
Variables		Chi-Square	df	*P-value*	
Day 7	0.03%	4.251	3	0.236	
	0.10%	3.304	3	0.347	
	0.30%	3.277	3	0.351	
	1.0%	4.889	3	0.180	
	3.0%	7.661	3	0.054	
	10%	4.452	3	0.217	
	30%	10.239	3	***0.017***	*

Variables		Chi-Square	df	*P-value*	
Day 14	0.03%	1.927	3	0.588	
	0.10%	5.010	3	0.171	
	0.30%	1.215	3	0.749	
	1.0%	0.437	3	0.932	
	3.0%	0.510	3	0.917	
	10%	2.056	3	0.561	
	30%	12.809	3	***0.005***	***

Post-hoc Analyses

Non-Parametric Pairwise Comparisons					
Day 7 - Sucrose 30%		Mann-Whitney U			
Compared Doses		MW-U	Z	*P-value*	
CTRL	LOW	5769.0	-1.240	0.215	
CTRL	MED	5253.0	-2.141	***0.032***	*
CTRL	HIGH	4161.0	-3.063	***0.002***	***
LOW	MED	5939.0	-0.940	0.347	
LOW	HIGH	4729.0	-1.929	0.054	
MED	HIGH	4836.0	-1.017	0.309	

Day 14 - Sucrose 30%		Mann-Whitney U			
Compared Doses		MW-U	Z	*P-value*	
CTRL	LOW	4982.0	-0.278	0.781	
CTRL	MED	3816.5	-2.901	***0.004***	***
CTRL	HIGH	4283.0	-2.218	***0.027***	*
LOW	MED	4152.0	-2.707	***0.007***	**
LOW	HIGH	4656.0	-1.999	***0.046***	*
MED	HIGH	4484.0	-0.745	0.456	

S1 Table. (C) No-Choice Experiment – data separated by dietary protein (all doses pooled)

(1) SURVIVAL

Kaplan-Meier Analyses

Overall Comparisons

	Chi-Square	df	*P-value*	
Log Rank (Mantel-Cox)	166.477	3	***<0.001***	***

Test of equality of survival distributions for the different levels of Diet.

Pairwise Comparisons

	Compared Diets		Chi-Square	*P-value*	
Log Rank (Mantel-Cox)	0:1	1:100	58.939	***<0.001***	***
	0:1	1:30	170.382	***<0.001***	***
	0:1	1:3	41.150	***<0.001***	***
	1:100	1:30	36.155	***<0.001***	***
	1:100	1:3	0.003	0.954	
	1:30	1:3	30.974	***<0.001***	***

Mann-Whitney Comparisons

Day 7		Mann-Whitney U			
Compared Diets		MW-U	Z	*P-value*	
0:1	1:100	954995.0	-5.469	***<0.001***	***
0:1	1:30	933945.5	-6.724	***<0.001***	***
0:1	1:3	967928.5	-4.155	***<0.001***	***
1:100	1:30	1027043.0	-1.280	0.201	
1:100	1:3	881421.0	-9.562	***<0.001***	***
1:30	1:3	860497.5	-10.786	***<0.001***	***

Day 14		Mann-Whitney U			
Compared Diets		MW-U	Z	*P-value*	
0:1	1:100	941693.5	-5.860	***<0.001***	***
0:1	1:30	810587.5	-12.541	***<0.001***	***
0:1	1:3	880573.0	-8.983	***<0.001***	***
1:100	1:30	916577.5	-6.805	***<0.001***	***
1:100	1:3	986805.5	-3.170	***0.002***	***
1:30	1:3	973930.0	-3.648	***<0.001***	***

S1 Table. (C) No-Choice Experiment – data separated by dietary protein (all doses pooled)

(2) NUTRITION

One-Way ANOVA

Test of Homogeneity of Variances				
**Variables**	**Levene Stat**	**df1**	**df2**	**Sig.**
Day 7	1.983	3	44	0.130
Day 14	5.037	3	44	***0.004***

ANOVA						
**Variables**		**Σ Squares**	**df**	**Mean Square**	**F**	**Sig.**
Day 7	Between Groups	56.385	3	18.795	0.659	0.582
	Within Groups	1255.476	44	28.534		
	Total	1311.861	47			
Day 14	Between Groups	612.792	3	204.264	2.518	***0.070***
	Within Groups	3569.578	44	81.127		
	Total	4182.371	47			

Note: because of non-homogenous variances on Day 14,
One-way ANOVA is not applicable here.

Kruskal-Wallis ANOVA

Non-Parametric Overall Comparison				
	**Chi-Square**	**df**	***P-value***	
Kruskal-Wallis on Day 14	8.485	3	***0.037***	*

Non-Parametric Pairwise Comparisons					
**Day 14**		**Mann-Whitney U**			
**Compared Diets**		**MW-U**	**Z**	***P-value***	
0:1	1:100	34.000	-2.194	***0.028***	*
0:1	1:30	32.000	-2.309	***0.021***	*
0:1	1:3	35.000	-2.136	***0.033***	*
1:100	1:30	50.000	-1.270	0.204	
1:100	1:3	55.000	-0.981	0.326	
1:30	1:3	67.000	-0.289	0.773	

S1 Table. (C) No-Choice Experiment – data separated by dietary protein (all doses pooled)

(3) SUCROSE RESPONSE

Kruskal-Wallis Analyses

Non-Parametric Overall Comparison					
Variables		Chi-Square	df	*P-value*	
Day 7	0.03%	4.384	3	0.223	
	0.10%	3.500	3	0.321	
	0.30%	25.273	3	***<0.001***	***
	1.0%	38.988	3	***<0.001***	***
	3.0%	11.357	3	***0.010***	**
	10%	4.724	3	0.193	
	30%	7.518	3	0.057	

Variables		Chi-Square	df	*P-value*	
Day 14	0.03%	2.435	3	0.487	
	0.10%	1.328	3	0.710	
	0.30%	9.582	3	***0.022***	*
	1.0%	23.527	3	***<0.001***	***
	3.0%	4.179	3	0.243	
	10%	2.326	3	0.507	
	30%	1.272	3	0.736	

Post-hoc Analyses (1/2)

Non-Parametric Pairwise Comparisons					
Day 7 - Sucrose 0.30%		Mann-Whitney U			
Compared Diets		MW-U	Z	*P-value*	
0:1	1:100	6053.0	-0.400	0.689	
0:1	1:30	4694.0	-3.991	***<0.001***	***
0:1	1:3	4961.0	-2.697	***0.007***	***
1:100	1:30	4958.5	-3.798	***<0.001***	***
1:100	1:3	5236.5	-2.427	***0.015***	*
1:30	1:3	4864.5	-1.491	0.136	

Day 7 - Sucrose 1.0%		Mann-Whitney U			
Compared Diets		MW-U	Z	*P-value*	
0:1	1:100	5293.5	-2.953	***0.003***	***
0:1	1:30	4440.0	-3.327	***0.001***	***
0:1	1:3	5419.5	-0.116	0.908	
1:100	1:30	3867.0	-6.004	***<0.001***	***
1:100	1:3	4986.5	-2.814	***0.005***	***
1:30	1:3	4119.0	-3.369	***0.001***	***

S1 Table. (C) No-Choice Experiment – data separated by dietary protein (all doses pooled)

(3) SUCROSE RESPONSE

Post-hoc Analyses (2/2)

Day 14 - Sucrose 0.30%		Mann-Whitney U			
Compared Diets		MW-U	Z	*P-value*	
0:1	1:100	4327.5	-1.778	0.075	
0:1	1:30	3998.5	-2.375	***0.018***	*
0:1	1:3	3721.5	-3.045	***0.002***	***
1:100	1:30	5111.0	-0.673	0.501	
1:100	1:3	4779.0	-1.431	0.152	
1:30	1:3	4777.5	-0.756	0.450	

Day 7 - Sucrose 3.0%		Mann-Whitney U			
Compared Diets		MW-U	Z	*P-value*	
0:1	1:100	6071.5	-0.082	0.935	
0:1	1:30	4528.0	-2.895	***0.004***	***
0:1	1:3	5021.5	-1.220	0.223	
1:100	1:30	4767.0	-2.851	***0.004***	***
1:100	1:3	5282.5	-1.154	0.249	
1:30	1:3	4651.0	-1.653	0.098	

Day 14 - Sucrose 1.0%		Mann-Whitney U			
Compared Diets		MW-U	Z	*P-value*	
0:1	1:100	4612.5	-0.399	0.690	
0:1	1:30	3940.5	-1.990	***0.047***	*
0:1	1:3	3289.5	-3.710	***<0.001***	***
1:100	1:30	4471.0	-2.483	***0.013***	*
1:100	1:3	3714.0	-4.267	***<0.001***	***
1:30	1:3	4356.5	-1.832	0.067	
