# Supplementary material for: Sucrose Sensitivity of Honey Bees Is Differently Affected by Dietary Protein and a Neonicotinoid Pesticide
Source: PLoS One. 2016 Jun 7;11(6):e0156584. doi: 10.1371/journal.pone.0156584 (PMC4896446; doi:10.1371/journal.pone.0156584)
Supplement: S2 Table — (PDF) [file pone.0156584.s003.pdf]

S2 Table. Spearman Correlations (underlined values in manuscript Tables 1 and 2)

(1) CHOICE EXPERIMENT

		Survival		Cumulative Consumption		PER Day 7		PER Day 14	
		Day 7	Day 14	Day 7	Day 14	10%	30%	10%	30%
	**Correlation Coefficient**	-0.102	-0.051	-0.124	0.097	0.003	0.054	-0.449	-0.364
**Dose**	***P-value***	0.634	0.812	0.564	0.652	0.990	0.801	***0.032***	0.088
	**N**	24	24	24	24	24	24	23	23

(2) NO-CHOICE EXPERIMENT

		Survival		Cumulative Consumption		PER Day 7		PER Day 14	
		Day 7	Day 14	Day 7	Day 14	10%	30%	10%	30%
	**Correlation Coefficient**	-0.290	-0.054	-0.141	-0.038	-0.195	-0.351	-0.063	-0.439
**Dose**	***P-value***	***0.046***	0.713	0.338	0.799	0.189	***0.016***	0.669	***0.002***
	**N**	48	48	48	48	47	47	48	48

		Survival		Cumulative Consumption		PER Day 7			PER Day 14		
		Day 7	Day 14	Day 7	Day 14	0.30%	1.0%	3.0%	0.30%	1.0%	3.0%
	**Correlation Coefficient**	-0.081	0.449	0.101	0.390	0.164	0.388	0.253	0.378	0.442	0.250
**Diet**	***P-value***	0.586	***0.001***	0.495	***0.006***	0.272	***0.007***	0.086	***0.008***	***0.002***	0.087
	**N**	48	48	48	48	47	47	47	48	48	48
