# Supplementary material for: Sucrose Sensitivity of Honey Bees Is Differently Affected by Dietary Protein and a Neonicotinoid Pesticide
Source: PLoS One. 2016 Jun 7;11(6):e0156584. doi: 10.1371/journal.pone.0156584 (PMC4896446; doi:10.1371/journal.pone.0156584)
Supplement: S3 Table — Every values are in mg/bee ±s.e.m., except for the P:C ratio columns. The “Cumulative Consumption” column is the same as the last column in Table 1. Honey bees were offered the choice between two unbalanced diets, differing in their P:C ratios (1:3 and 1:30). First, consumption of each diet is assessed and proportions of protein and carbohydrate are calculated. The total protein consumption is the addition of the protein parts eaten from the 1:3 diet and the 1:30 diet; the same goes for the carbohydrate part. P:C ratios are calculated by dividing the total protein consumption by the total carbohydrate consumption. (PDF) [file pone.0156584.s004.pdf]

S3 Table. Detail of cumulative protein and carbohydrate intakes during the Choice Experiment after 7 and 14 days.

Experience		Cumulative Consumption	Diet P:C 1:3		Diet P:C 1:30		Total Protein Consumption	Total Carbs Consumption	P:C ratio	s.e.m.
			P	C	P	C				
Day 7	TOTAL	65.3 ±0.8	8.0 ±0.1	24.1 ±0.4	1.07 ±0.02	32.0 ±0.5	9.1 ±0.2	56.1 ±0.7	**1 : 6.16**	[1 : 6.34 - 1 : 5.99]
	CTRL	64.8 ±1.8	7.9 ±0.4	23.8 ±1.2	1.06 ±0.03	31.9 ±0.9	9.0 ±0.4	55.8 ±1.5	**1 : 6.19**	[1 : 6.64 - 1 : 5.78]
	LOW	67.0 ±1.4	8.4 ±0.3	25.2 ±0.9	1.08 ±0.03	32.3 ±0.8	9.5 ±0.3	57.5 ±1.2	**1 : 6.07**	[1 : 6.40 - 1 : 5.77]
	MED	65.0 ±1.4	8.0 ±0.2	23.9 ±0.7	1.07 ±0.02	32.1 ±0.7	9.0 ±0.2	56.0 ±1.1	**1 : 6.19**	[1 : 6.49 - 1 : 5.91]
	HIGH	64.2 ±1.9	7.9 ±0.3	23.6 ±0.8	1.06 ±0.05	31.7 ±1.4	8.9 ±0.3	55.3 ±1.7	**1 : 6.20**	[1 : 6.61 - 1 : 5.82]
Day 14	TOTAL	134.2 ±2.1	17.0 ±0.4	50.9 ±1.1	2.14 ±0.05	64.2 ±1.5	19.1 ±0.4	115.1 ±1.9	**1 : 6.03**	[1 : 6.25 - 1 : 5.81]
	CTRL	130.3 ±2.6	16.2 ±0.6	48.7 ±1.7	2.11 ±0.04	63.3 ±1.1	18.3 ±0.6	112.0 ±2.1	**1 : 6.10**	[1 : 6.42 - 1 : 5.81]
	LOW	138.9 ±4.1	17.8 ±1	53.3 ±3.1	2.19 ±0.05	65.6 ±1.5	20.0 ±1.0	118.9 ±3.2	**1 : 5.96**	[1 : 6.45 - 1 : 5.52]
	MED	130.1 ±3.9	16.4 ±0.5	49.1 ±1.4	2.09 ±0.10	62.6 ±2.9	18.4 ±0.5	111.6 ±3.5	**1 : 6.06**	[1 : 6.42 - 1 : 5.71]
	HIGH	137.5 ±5.7	17.5 ±0.8	52.4 ±2.4	2.18 ±0.17	65.4 ±5.1	19.7 ±0.8	117.9 ±5.3	**1 : 6.00**	[1 : 6.53 - 1 : 5.50]

Cumulative consumption and details of the different amount of nutrient eaten during the Choice Experiment. Every values are in **mg/bee ±s.e.m.**, except for the P:C ratio columns. The “Cumulative Consumption” column is the same as the last column in Table 1. Honey bees were offered the choice between two unbalanced diets, differing in their P:C ratios (1:3 and 1:30). First, consumption of each diet is assessed and proportions of protein and carbohydrate are calculated. The total protein consumption is the addition of the protein parts eaten from the 1:3 diet and the 1:30 diet; the same goes for the carbohydrate part. P:C ratios are calculated by dividing the total protein consumption by the total carbohydrate consumption.
